# A qualitative study exploring Malaysian women’s preferences for authority or emotional appeals in public health messages promoting breast cancer screening

**DOI:** 10.1038/s41598-025-31687-7

**Published:** 2026-01-12

**Authors:** Nicholas Yee Liang Hing, Pei Xuan Kuan, Emma Mirza Wati Mohamad, Ching Ee Loo, Komathi Perialathan, Aimi Nadiah Mohamad Norzlen, Yan Yee Yip, Wan Mohd Shariffuddin Zainudin, Shazimah Abdul Samad, Zakiah Mohd Said, Wen Yea Hwong

**Affiliations:** 1 Institute for Clinical Research, National Institutes of Health, Ministry of Health, Block B4, Jalan Setia Murni U13/52, Seksyen U13, Setia Alam, 40170 Shah Alam, Selangor Malaysia; 2 Faculty of Social Sciences and Humanities, Universiti Kebangsaan Malaysia, Bangi, Selangor Malaysia; 3 Institute for Health Behavioural Research, National Institutes of Health, Ministry of Health, Shah Alam, Selangor Malaysia; 4 Family Health Development Division, Ministry of Health, Putrajaya, Malaysia

**Keywords:** Health communication, Authority appeal, Emotional appeals, Breast cancer screening, In-depth interview, Public health, Cancer, Health care, Medical humanities, Psychology, Psychology

## Abstract

Persuasive health communication is key to promoting breast cancer screening. Due to existing gaps over how public health messages should be framed, a study was conducted to explore the preferences and perceptions of Malaysian women towards messages that incorporate different types of appeals to promote breast cancer screening. In-depth qualitative interviews were conducted among Malaysian women aged 40 and above with no previous history of breast cancer or other cancers. They were exposed to messages that promoted breast cancer screening using seven different appeals; authority, hope, social, humour, fear, sadness, and shame-guilt. Qualitative expressions for preferences and perceptions were analysed using interpretive thematic analysis. 41 Malaysian women were interviewed. The majority preferred messages that incorporated hope, authority, or fear appeals. Messages with an authority appeal were favoured for their personalised and authoritative effect. Messages using hope and fear appeals elicited emotional reactions and cognitive appraisals that contributed to their favourability. In contrast, humour, sadness, and shame-guilt were the most disliked appeals. The humour appeal was generally disliked because it caused interpretations that weakened the persuasive impact of the advocated behaviour in the message. Messages incorporating sadness and shame-guilt appeals were disliked primarily due to their negative framing. In conclusion, public health messages incorporating hope, authority, or fear appeals show promising potential to motivate breast cancer screening among Malaysian women, and their effectiveness in influencing actual behaviour change should be further explored.

**Supplementary Information:**

The online version contains supplementary material available at 10.1038/s41598-025-31687-7.

## Introduction

Breast cancer is the most common cancer in Malaysia, with as high as 17.6% of all malignancies reported. Most breast cancer cases are detected at an advanced stage, with increasing trends from 43.2% in 2007 to 2011, 48.0% in 2012 to 2016, and 50.5% in 2017 to 2021^1^. Early detection and diagnosis of breast cancer are crucial to improve prognostic outcomes^2^. This is achievable by attending regular breast cancer screening procedures, such as clinical breast examinations (CBE) and mammograms^3^.

Alarmingly, breast cancer screening uptake rates in Malaysia remain poor. Data from the National Health Informatics Centre have shown low participation rates for CBE at healthcare clinics, with an attendance rate of less than 30%^4^. Several studies found poor uptake of mammograms among eligible populations, including high-risk women^5,6^. The National Health & Morbidity Survey (NHMS) reported that only 25% of women had ever undergone a mammogram, and only 21% of women aged 40 and above had a mammogram within the past three years^4^.

Given these low screening rates, public health strategies directed at motivating Malaysian women to attend screening should be employed. Persuasive health communication is one key strategy that leverages tailored messaging to influence attitudes and behaviours. By designing messages that resonate with the target audience, persuasive health communication provides the capacity to strengthen facilitators and address barriers to a target behaviour, subsequently improving the likelihood of behaviour change, such as attending breast cancer screening^7,8^.

Multiple techniques are available to design persuasive health communication. A fundamental element lies with framing communicated content to arouse emotions and trigger cognitive appraisals necessary to motivate intended behaviour changes. This can be achieved by incorporating emotional appeals to elicit affective responses that drive decision-making. Aligned with the concept of affect heuristics, this strategy capitalises on how people use their immediate emotional responses to guide their judgments and actions^9^. This method of strategic framing is widely employed in the advertising industry where emotional appeals are incorporated to make content compelling and enticing to customers^10^.

Emotional appeals exist in both polarities. Fear and sadness appeals are commonly used to arouse negative emotions by highlighting consequences of engaging in negative behaviour^11,12^. Similarly, shame-guilt appeals arouse negative emotions by emphasizing the moral, social, or ethical inappropriateness of certain behaviours, resulting in consequences not only for oneself but also for significant others^13^. Conversely, hope appeals emphasize positive outcomes associated with recommended behaviours to arouse positive emotions^14^. Humour is also a positive appeal, but uniquely acts by invoking a sense of absurdity to elicit amused expressions to draw attention and raise salience^15^. Social appeal is another positive emotional appeal that emphasises social influences and relationships to influence behaviour. It leverages various types of social elements such as social norms, social responsibility, or social acceptance^16,17^.

Persuasive communication can also exist without emotional appeals. Messages incorporating an authority appeal leverage on a social influence principle called the “authority principle” where individuals comply with recommendations from respectable authority figures or credible sources^18^. Healthcare professionals and established institutions are viewed as credible bodies of authority, which helps instil trust and encourage adoption of advocated health-promoting behaviours, as reported by several studies^19–21^.

Although the use of various appeals to promote breast cancer screening have been studied^15,22–24^, evidence specific to certain emotional appeals, such as social and sadness appeals, are limited^25^. There is also a paucity of evidence in Malaysia. Most studies have been conducted outside the Southeast Asian region. As perception and behaviour are shaped by context and culture, findings from vastly different settings are less applicable to the Malaysian population. Recognising the opportunity to generate evidence that could guide both local and global health communication designers in framing effective public health messages, a study was conducted to explore the preferences and perceptions of Malaysian women towards breast cancer screening promotional messages that incorporate seven different appeals; authority, hope, social, humour, fear, sadness, and shame-guilt.

## Methods

### Study design and setting

To gain a deep understanding of how public health messages were interpreted, in-depth qualitative interviews were conducted among recruited participants. These interviews took place in the Klang Valley, a metropolitan region in Malaysia. It encompasses the federal territories of Kuala Lumpur and Putrajaya, together with their surrounding cities and towns located within the state of Selangor. Predominantly urban, the region is characterised by diverse cultural backgrounds arising from a multi-ethnic population, composed mainly of Malays, Chinese, and Indians. Klang Valley is home to individuals from various socioeconomic backgrounds, providing a platform to explore a wide range of perspectives, making it an ideal setting for studying public health messages.

To ensure participants felt comfortable and could freely express their opinions, the interviews were conducted in an enclosed location that provides a conducive and private environment.

### Participants selection

Malaysian females aged 40 and above with no previous history of breast cancer or other cancers were recruited into our study. Participants were purposively sampled based on guidance drawn from a sampling matrix (Supplementary Table 2) that accounted for the three major ethnicities in Malaysia (i.e. Malay, Chinese, and Indian), with different education levels (i.e. secondary education level and below, and above secondary education level) and breast cancer screening practices (i.e., those who have and have not undergone a CBE or mammogram within the recommended time periods of one and two years, respectively) in order to provide an inclusive scope of information that encompasses diverse perspectives. By assigning four women per cell in the matrix, the estimated sample size was 48 women. Potential participants were identified through purposive sampling of women who fulfilled inclusion criteria.

### Study instruments

Volunteers from two non-governmental organisations advocating for breast cancer awareness were first involved in our study during the pre-design phase of the tested messages. Ideas were solicited for message development based on the seven specific appeals that were identified from the literature with consensus among the study team.

Gathered ideas formed the foundation of the actual design of the tested messages. Experimental short text-based messages that promoted breast cancer screening were co-designed with local health promotion practitioners and a health communication researcher to ensure they communicate each appeal effectively, resonating with the local culture. To maintain consistency and control for other parameters that may affect preference and perceptions, designed messages only had three to four sentences, with approximately similar total number of words in each message. The last sentence was standardised across all tested messages with an identical call to action.

Given that there are multiple ways to frame a message within a specific appeal, we initially designed three messages for each appeal to help elicit preferences in a broader manner. However, when we piloted the messages, respondents felt overwhelmed to filter too many messages. Finally, we decided to only design two messages for each appeal, resulting in 14 messages.

The authority messages were constructed with the goal of providing an authoritative voice that instructed eligible women to attend screening. To enhance the authoritative tone, the messages were fitted with the age group that specifically addressed eligible women.

Hope appeal messages were centred on highlighting the benefits of early breast cancer detection through screening, which offers hope for improved survival.

Various ways exist to incorporate social appeals. Hence, the study team reached a consensus to design social appeal messages that emphasise social connectedness among individuals with the broader community to achieve a common societal goal of promoting breast cancer screening.

Humour appeal messages were designed to be humorous with punchlines, creating an overall light and entertaining tone.

Hard statistics and threatening statements were incorporated into the fear appeal messages to emphasise the potential dangers associated with late breast cancer detection.

Based on recommendations by Leek et al. to reference family and friends in mammography-related advertising^26^, we used loved ones and family-themed constructs in our messages incorporating sadness and shame-guilt appeals.

Designed messages were piloted with ten women from the public to ensure that they were comprehensible and conveyed the intended appeal. Responses obtained were used to refine the messages, which were later finalised after several iterations. Finalised messages were subjected to a forward and backward translation to produce messages in Bahasa Malaysia, Malaysia’s national language. These were content validated by a language expert to ensure they conveyed a similar meaning with the English version, before being piloted again with a different set of ten women. Table 1 presents all the finalised messages.

**Table 1:** Tested messages with the corresponding incorporated appeals.

Type of appeal	Message no.	Message
Authority	1	The Malaysian Ministry of Health urges all women 40 years old and above to get themselves checked for breast cancer every two years. Don’t delay. Get your screening appointment today.
	2	Are you 40 years old and above? If yes, doctors recommend that you should get checked for breast cancer every 2 years. Take action! Get your screening appointment today.
Hope	1	9 out of 10 women can survive breast cancer if found and treated early. Your chances are brighter with early screening. Get your screening appointment today.
	2	Every breast cancer screening carries hope to beat breast cancer. Early detection saves lives. Get your screening appointment today.
Social	1	Going for breast cancer screening will encourage women around you to do the same. Be an example to them. Get your screening appointment today.
	2	Come join all Malaysian women to fight against breast cancer. Take the first step by screening for breast cancer. Get your screening appointment today.
Humour	1	It’s hard to decide what clothes to wear. Luckily you don’t need to choose which breast needs to be screened. Get your screening appointment today.
	2	Breasts can’t speak up to ask for help. Luckily doctors can understand breast language when they conduct breast cancer screening. Get your screening appointment today.
Fear	1	Breast cancer kills more than 3500 Malaysian women every year. If breast cancer finds you too late, you might become one of them. Don’t delay. Get your screening appointment today.
	2	Being diagnosed with late-stage breast cancer is scary. You may not have much hope for survival. Get your screening appointment today.
Sadness	1	Your family will cry when they see you suffer from late-stage breast cancer. Don’t let that happen. Get your screening appointment today.
	2	Saying goodbye too soon because of breast cancer is heartbreaking. Avoid experiencing such sadness. Get your screening appointment today.
Shame-guilt	1	Late breast cancer detection lets your loved ones down. Wouldn’t it be a shame if that happened to you? Get your screening appointment today.
	2	Lives of loved ones will be ruined if you have late- stage breast cancer. Are you willing to see them suffer? Get your screening appointment today.

### Study procedure

In-depth interviews were conducted on identified participants. Participants were briefed, consented, and given the 14 investigated messages in a random order and provided sufficient time to read them through. After that, participants were prompted to select messages that they liked or found motivating for attending breast cancer screening. A message that was selected by a participant counted as one vote of preference for that message. For each selected message, participants were asked if it evoked any form of emotions before asking them to freely express reasons that led them to shortlist the message. This entire process was repeated for messages that participants disliked or felt demotivating. All interviews were recorded using an audio recorder.

Data collection period lasted for three months, i.e. July to October 2023. Participant recruitment was halted upon reaching saturation. A detailed version of the study procedure is presented in the supplementary materials.

### Ethics declaration

This study has been registered with the Malaysian National Medical Research Register (NMRR ID-22-02616-I2I) and received ethical approval from the Medical Research and Ethics Committee of the Ministry of Health Malaysia (Ref: 22-02616-I2I). The study was conducted in compliance with ethical principles outlined in the Declaration of Helsinki and Malaysian Good Clinical Practice Guideline.

### Data analysis

Recorded interviews were transcribed verbatim. Transcripts were cross-checked among transcribers to ensure data accuracy. Characteristics of preferred and disliked appeals were analysed and coded independently using interpretive thematic analysis by NYLH and PXK. Codes and analyses were then cross-checked to ensure consistency and objectivity. CEL was consulted to resolve any disputed codes or themes that could not be unanimously agreed upon. Findings were further consolidated and finalised among study team members through several discussions. Selected excerpts were de-identified to preserve anonymity.

We determined which appeals were preferred or disliked among our interviewees by tabulating votes for both preferred and disliked messages. If at least one of the two messages for an appeal is voted, that appeal would receive a vote. This vote tabulation exercise was not intended to produce generalisable data, but rather to complement the thematic exploration by highlighting which message appeals were the most preferred or disliked among our sample.

## Results

41 women comprising an almost equal representation of all three major ethnicities in Malaysia were interviewed. Approximately half of the participants were aged 50 years and above, possessed intermediate to high education levels, and have either undergone a clinical breast examination in the past year or a mammogram within the last two years. Table 2 presents the characteristics of the interviewed participants.

Most participants preferred messages with a hope or authority appeal to motivate breast cancer screening. A considerable number also preferred messages with a fear appeal. Meanwhile, humour and shame-guilt received the fewest votes, indicating the lowest level of preference among participants.

Conversely, humour, sadness, and shame-guilt appeals were the most disliked for motivating breast cancer screening. Supplementary Table 1 presents a tabulation of votes for both preferred and disliked appeals.

**Fig. 1:**
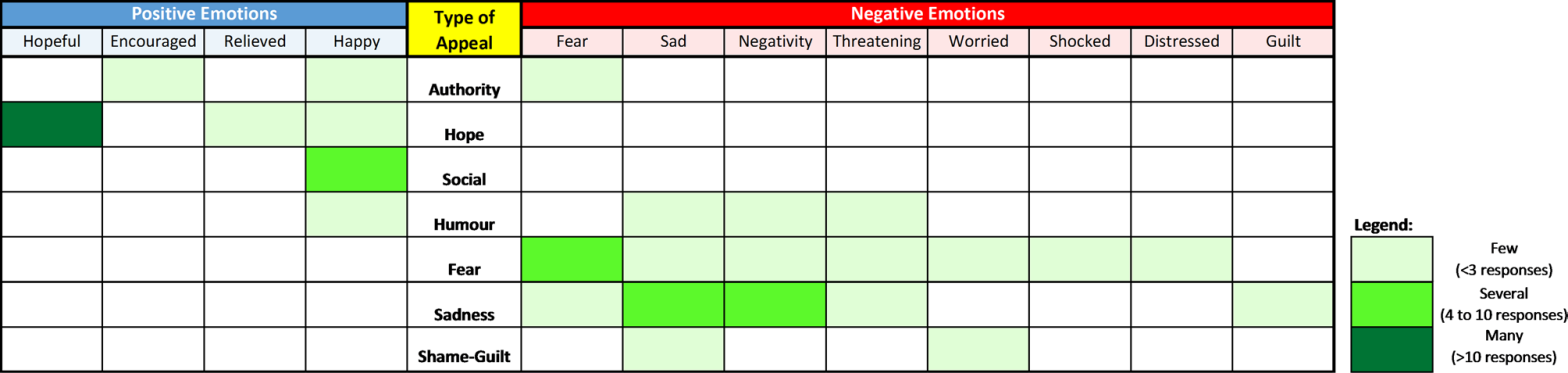
Type of emotions evoked from participants through each of the investigated appeals. Frequency of an emotion was based on the highest response received from one of the two tested messages for each appeal.

Figure 1 presents the type of emotions elicited through the investigated messages. Emotional responses were varied. Some participants exhibited strong reactions while others showed minimal or no emotional response. Messages applying an emotional appeal generally evoked emotions that corresponded with the valence of the appeal, albeit at varying degrees with slight nuances. The hope appeal messages invoked one of the strongest positive emotional responses. The authority appeal messages, although being emotionally neutral, aroused bivalent emotional responses.

**Fig. 2:**
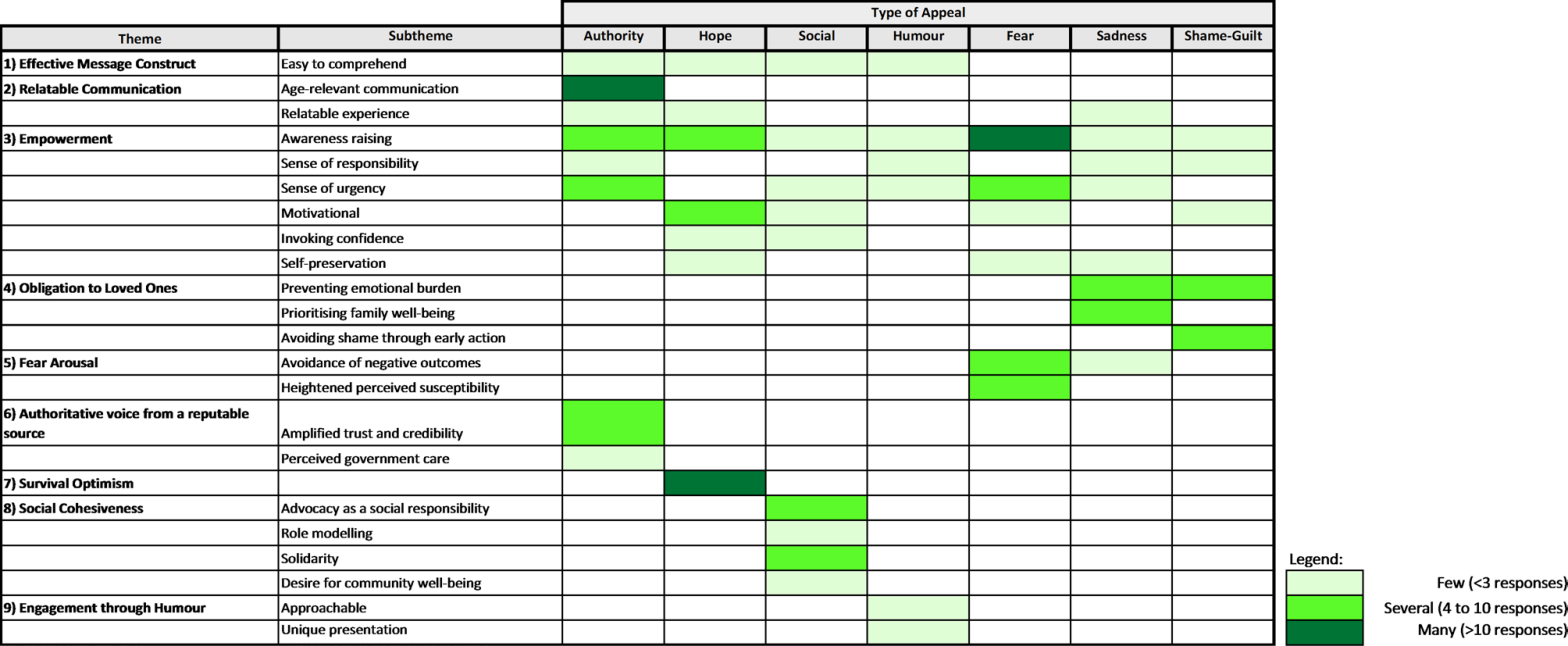
Themes and subthemes that emerged from each tested appeal for preferred messages. Frequency of a theme or subtheme was based on the highest response received from one of the two tested messages for each appeal.

**Fig. 3:**
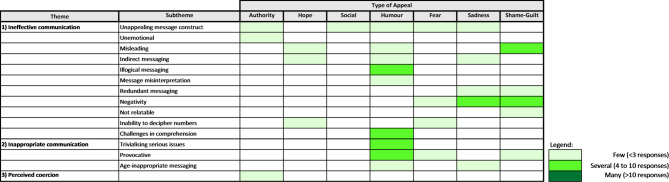
Themes and subthemes that emerged from each tested appeal for disliked messages. Frequency of a theme or subtheme was based on the highest response received from one of the two tested messages for each appeal.

Figures 2 and 3 summarises thematic responses from each of the investigated appeals that were preferred and disliked, respectively. Descriptions of coded themes and subthemes are presented in the supplementary material.

Overall, several participants highlighted that they preferred certain messages because they were easy to understand. The ease of comprehension stems from the message being simple, clear, and direct to the point. For example, through the Hope 1 message, a few participants found that the use of numbers made the message clearer and more direct, without needing to interpret words.

All the studied appeals raised awareness on breast cancer screening to various degrees. Awareness was raised either by highlighting the age group requiring screening, the importance of screening, the consequences of not attending it, or by presenting meaningful facts. Most of the appeals also conferred a sense of responsibility or sense of urgency to screen for breast cancer at varying levels. Urgency arose from either a fear response, a recommendation from an authoritative figure, or awareness of the age group requiring screening.

The following section explains each appeal in order from most to least preferred, highlighting the themes and subthemes that stood out for each.

**Table 2:** Characteristics of interviewed participants.

Subject no.	Ethnicity	Age	Highest education level	Occupation	Marital status	Screening status
1	Malay	43	Diploma	Customer Service Officer	Married	No
2	Malay	59	Malaysian Certificate of Education	Security Guard	Widow	No
3	Malay	46	Malaysian Certificate of Education	Janitor	Married	No
4	Malay	43	Malaysian Certificate of Education	Clerk	Married	No
5	Malay	55	Bachelor’s Degree	Engineer	Divorced	Yes
6	Malay	55	Bachelor’s Degree	Banker	Married	Yes
7	Malay	42	Polytechnic Certificate	Clerk	Single	No
8	Malay	41	Diploma	Secretary	Married	No
9	Chinese	48	Diploma	Housewife	Married	No
10	Chinese	43	Bachelor’s Degree	Housewife	Married	Yes
11	Chinese	49	Diploma	Remisier	Married	Yes
12	Chinese	52	Diploma	Self-employed	Married	Yes
13	Chinese	58	Masters	Teacher	Single	No
14	Chinese	53	Bachelor’s Degree	Housewife	Married	No
15	Indian	46	Malaysian Certificate of Education	Janitor	Widow	No
16	Indian	44	Lower Secondary Assessment	Janitor	Married	No
17	Indian	66	Diploma	Administrator	Married	No
18	Indian	71	Bachelor’s Degree	Doctor	Married	No
19	Indian	47	Masters	Clinical Trial Coordinator	Single	No
20	Malay	59	Malaysian Certificate of Education	Housewife	Married	No
21	Malay	45	Malaysian Certificate of Education	Dental Surgery Assistant	Married	Yes
22	Malay	56	Diploma	Manager	Married	No
23	Chinese	45	Malaysian Certificate of Education	Housewife	Married	Yes
24	Chinese	41	Diploma	Housewife	Married	No
25	Chinese	52	Bachelor’s Degree	Manager	Single	Yes
26	Indian	48	Bachelor’s Degree	School Teacher	Married	No
27	Indian	54	Malaysian Certificate of Education	Unemployed	Divorced	Yes
28	Indian	41	Bachelor’s Degree	Doctor	Married	Yes
29	Chinese	66	Malaysian Certificate of Education	Retiree	Married	No
30	Chinese	50	Malaysian Certificate of Education	Accounts Executive	Married	No
31	Chinese	57	Malaysian Certificate of Education	Sales Executive	Divorced	No
32	Indian	42	Diploma	Interpreter	Divorced	No
33	Malay	51	Malaysian Lower Certificate of Education	Housewife	Married	Yes
34	Indian	41	Malaysian Certificate of Education	Ayurveda Practitioner	Divorced	Yes
35	Chinese	44	Malaysian Certificate of Education	School Teacher	Divorced	Yes
36	Malay	45	Malaysian Certificate of Education	Housewife	Married	Yes
37	Malay	40	Bachelor’s Degree	Self-employed	Divorced	Yes
38	Chinese	48	Malaysian Certificate of Education	School Teacher	Married	Yes
39	Chinese	69	Malaysian Certificate of Education	Retiree	Single	Yes
40	Indian	54	Diploma	Director	Married	Yes
41	Indian	51	Malaysian Certificate of Education	Housewife	Married	No

### Hope

A large number of participants preferred the hope appeal messages because they provided an optimistic sense of survival. The messages encouraged optimism by reassuring individuals that early cancer detection through screening can lead to successful treatment outcomes. This was achieved either with the use of statistics or a general statement regarding survivability associated with early detection.

“So in a way, it’s like giving you hope. So it feels hopeful…it’s not really. you know, a death sentence per se because, because if you think about cancer, its like…like a death sentence right? So, when you say that 9 out of 10 women can survive, that’s quite a good statistic.”

**(Hope 1 | Subject 6)**

Additionally, with the messages arousing positive feelings, several participants expressed motivation to act. A few participants were also motivated in response to these messages arousing an attitude of self-preservation and providing confidence to act.

### Authority

Messages with an authority appeal were preferred mainly due to reasons categorised under the relatable communication theme. Messages which aligned with specific characteristics, experiences, and contexts that are relevant to the audience helped elicit favourable responses.

Age-relevant communication was the most prominent subtheme arising from a large majority of participants. The highlighted age group in these messages made it sound personal to them because they were in the same age category. This made them feel that the message was addressing them, compelling them to attend screening.

“So…this message is like… you know, personally to me…You know like… like addressing me like that. Okay now, I’m forty years and above and I should go for the screening.”

**(Authority 2 | Subject 13)**

Displaying the age group also made a few participants recall relatable experiences regarding how people from their social circle were diagnosed with breast cancer or died from the disease at a similar age. This enhanced the resonance of the message, increasing the salience of the communicated content.

The Authority 1 message yielded responses associated with the use of an authoritative figure. Authoritative voice from a reputable source was a major theme derived from this message. It explores how a credible and authoritative body can significantly impact the decision or perception of individuals towards a prescribed health-related behaviour.

Several participants emphasized how the presence of the Malaysian Ministry of Health (MOH), a credible source in the message, not only strengthens its impact but also enhances the audience’s trust in the information being shared. It highlights the role of the source in building confidence and reliability, making the prescribed action more likely to be accepted and acted upon.

A few participants expressed appreciation that the government initiates proactive efforts to ensure the well-being of the population, perceiving that the government cares for Malaysians. Participants were observed expressing appreciation via three aspects; the perception that the government is taking proactive measures for the people’s welfare, having an assurance of support from the government to the layperson, and providing advice that goes beyond race.

“Because the Ministry is putting in the effort, they are urging everybody to go get checked, that is very important.”

**(Authority 1 | Subject 41)**

### Fear

The fear appeal messages aroused the most diverse range of negative emotions. Fear arousal was a central theme emerging from responses captured through the fear appeal messages. The messages invoked fear to promote an emotional and cognitive response that motivated breast cancer screening.

Several participants liked the Fear 2 message because it provoked fear about the negative outcomes associated with late-stage breast cancer. This fear fostered intentions of attending breast cancer screening to avoid negative outcomes associated with premature death. A few participants also shared similar views through the Fear 1 message.

“Okay because this one is making me alert of this cancer and making me scared if it becomes late, we cannot do anything, so it’s giving me a hint to do screening.”

**(Fear 2 | Subject 26)**

Several participants expressed fear from the perceived susceptibility of breast cancer after noticing the mortality rate in the Fear 1 message. By emphasizing the likelihood that they or their family members could be affected, the message increases concern and prompt proactive health behaviours.

“I did think whether I will become one of the 3,500 people.”

**(Fear 1 | Subject 15)**

Several participants explained that the fear response stimulates an urgency to attend breast cancer screening.

“They will act faster to get screening programme, screening appointment. They are scared because of the such big amount.”

**(Fear 1 | Subject 38**).

Apart from arousing fear, awareness was raised regarding the consequences of late-stage breast cancer to a large number of participants, particularly through the Fear 1 message which presented an eye-catching mortality rate.

### Sadness

Obligation to loved ones was a central theme arising from participants who favoured the sadness appeal messages. The desire to prevent emotional burden, such as emotional distress or disappointment, from occurring to their family due to their personal inaction to detect cancer early was especially apparent through the Sadness 1 message.

“I don’t want them to see me suffer you know, I don’t want them to cry. So, I don’t want this thing to happen, so definitely you know, I have to go and get it checked, that kind of emotional impact.”

**(Sadness 1 | Subject 34)**

Several women reading this message also expressed a strong sense of duty to ensure the well-being of their family by making sure they are protected from late-stage cancer. This expression comes amidst the perception that family members, especially young children, were dependent on the participant’s care and support.

“….ah, family because family is a priority, right? Because I have many, there are still many school-going children. I have to look after, have to be there for them.”

**(Sadness 1 | Subject 36)**

However, a substantial number of responses indicated that this appeal was ineffective in persuasively communicating the advocated behaviour. Several participants disliked these messages because they conveyed an excessively negative vibe associated with sadness, death, fear, guilt, or feeling threatened. According to some participants, negativity fails to motivate and trigger a positive response for behaviour change.

A few participants also commented that the Sadness 1 message narrated an obvious situation about family grieving if a member was diagnosed with cancer, hence making the content redundant.

### Social

A few participants highlighted how the social appeals messages resonated with them because they emphasise the role of individuals in advocating for cancer screening within their communities. The messages confer a sense of collective responsibility to educate and encourage community members to screen.

“Yes, because many of the women they don’t know, they still haven’t went for this mammogram programme, so encourage women around you to do the same. This is important. To get awareness from all the women.”

**(Social 1 | Subject 38)**

A sense of solidarity was aroused among several women through the Social 2 message. They expressed a willingness to stand together and be unified to achieve a common goal of fighting against breast cancer.

“Ya, this one. I like it also because it’s like we’re all united in…fighting breast cancer. So part of the…the force.”

**(Social 2 | Subject 18)**

The Social 1 message garnered favourability from a few participants because it conveys the idea of individuals being role models to inspire women around them to attend breast cancer screening.

“Yes, that is all about it. The encouragement, be an example, be the champion for all the women out there. Yeah, that you are.”

**(Social 1 | Subject 22)**

### Humour

The humour messages drew significantly more negative criticisms as compared to positive responses. A few participants disliked the humour messages because the embedded humour added a layer of interpretation, making them indirect. A few participants with lower education levels misinterpreted the meaning behind the Humour 1 message, causing them to respond in an incoherent manner.

Several participants found the humour messages illogical, as they were perceived to lack logical flow or relevance to the promoted subject. This perception mainly stemmed from the failure to appreciate the humour of the message, or interpreting the message in a literal sense. For instance, in the Humour 2 message, some participants complained about the absurdity of mentioning a doctor understanding “breast language”, pointing out that breast cannot speak or it is not humanly possible, as doctors rely on machines to screen the breast. Some criticised the logic of relating the issue of choosing what clothes to wear with breast cancer screening in the Humour 1 message.

“Like breast cancer with clothes, what’s the connection? None, no connection at all, so I really don’t like this kind of message.”

**(Humour 1 | Subject 15)**

Several participants felt that the humour appeal caused messages to be perceived as making light of a serious health matter, making them less likely to be taken seriously and reducing their motivational value.

“Because I feel like it’s not giving a seriousness in the message. It’s more like funny, so I feel like it’s not the proper way of encouraging women to do screening.”

**(Humour 2 | Subject 26)**

Several participants also complained that these messages were provocative, drawing criticisms that invoked negative sentiments or risked being misinterpreted as insensitive by the opposite gender. For instance, by associating breast cancer screening with women being fickle-minded about what clothes to wear, a few participants were annoyed with the Humour 1 message as they felt it made fun of women.

“You are like rubbing on my nose, you know. I know that it’s actually like it’s…I need to go screening but like putting in clothes I’m wearing and then emphasizing on my breasts. That kind of things, that is a put me off.”

**(Humour 1 | Subject 22)**

### Shame-guilt

Participants favouring the shame-guilt appeal messages provided responses that resonated with the theme of having obligations to loved ones. Several participants expressed the desire of preventing emotional burden happening to their family through both messages.

“Go and get screened motivation because you do not want to let your loved ones down. You make sure that you are healthy, you make sure you’re okay.”

**(Shame-guilt 2 | Subject 17)**

The Shame-guilt 1 message triggered several responses that emphasised on the motivation to screen for breast cancer to prevent future shame or regret when facing family members with an avoidable calamity.

Ya, this one about family, and after they said, why didn’t you go? Why didn’t you go for breast cancer screening you know… Correct? Your family member will sure say that, right? You should have done it earlier.”

**(Shame-guilt 1 | Subject 13)**

Participants perceiving the shame-guilt appeal messages negatively mostly provided comments that were categorised under the ineffective communication theme. Similar to the sadness appeal, several participants disliked these messages for framing the content negatively with elements of sadness, regret, or a sense of family life being ruined. Several participants also felt that the messages were misleading because of discrepancies from their experienced realities. Based on past experience, they argued that there is always a chance that breast cancer goes undetected despite attending regular screening.

Other examples of misleading messages were quoted in the Shame-guilt 1 message, where a participant highlighted that instead of feeling let down, family members are, in fact, the ones who are supportive throughout the patient’s cancer journey.

“The woman is supposed to be the first person to feel let down, talk about them first, they (loved ones) will be supportive when she is sick, not let down.”

**(Shame guilt 1 | Subject 41)**

The shame-guilt messages framed women with late-stage breast cancer as the cause of their family’s suffering or hardship, which caused a few participants to feel hurt and anger. They felt these messages were being provocative by either victimising women who were already suffering but still required to think of their family’s well-being, or blaming them for an event that was beyond their control, even if they attended screening.

A few participants disliked the Shame-guilt 2 message because it feels unrelatable to themselves or to other people. For example, one participant mentioned that she believes the lives of loved ones will not be ruined and would still go on after a woman dies of breast cancer. Another participant explained that the message will not be applicable to those who are single or having a troubled relationship with their family.

## Discussion

Qualitative insights revealed that the emotional reactions to messages with emotional appeals were weaker than expected. This may be due to the messages being fully textual, which could have limited their ability to evoke strong emotional engagement. Nevertheless, the hope and fear appeal messages elicited more emotional reactions compared to other appeals. This may have triggered cognitive responses that contributed to their favourability among most participants. Interestingly, despite being emotionally neutral in nature, messages incorporating an authority appeal were largely favoured as they conferred a personalised and authoritative effect to motivate action. In contrast, the perceived negativity through the sadness and shame-guilt appeal messages, and the inability to appreciate the humour embedded in the humour appeal messages, caused these appeals to be the most disliked.

By highlighting the age group eligible for breast cancer screening in the authority messages, the messages inadvertently introduced a tailoring element, as they were framed in a context that matched participants’ attributes and therefore felt personally relevant to them^27^. Tailoring in a contextual manner enhances cognitive preconditions for message processing by increasing attention to aid comprehension and recall, and stimulating deeper processing of information for greater persuasive impact^27^. This form of tailoring also encourages self-referential thinking, prompting individuals to relate to their own personal situations and experiences. These thought processes could explain the emergence of fear amongst a few participants, along with the other themes and subthemes elicited through the authoritative messages.

The use of authoritative personas conferred favourability to the authority appeal messages by fostering greater trust and credibility in the promoted content. This suggests that medical professionals and the Malaysian Ministry of Health possess a considerable degree of authority bias among the public, which enhances the perceived reliability of communicated messages. Our findings concur with previous studies that demonstrate an enhanced perceived credibility or trustworthiness of communicated content through the use of authoritative figures to significantly influence acceptance of a recommendation^28^. For instance, research on vaccine advocacy has shown that individuals are more likely to consider vaccination when the message comes from healthcare providers, who are viewed as highly credible and knowledgeable^29^. An additional spillover effect worth highlighting is the positive emotions arising from perceiving that the government is responsible and cares for the people. Despite all these positive influences, it is crucial to balance authority with approachability to avoid perceptions of coercion or feeling pressured, which can counteract the intended effects^30^.

The hope appeal messages enhanced the perceived benefits of screening, which contributes to the appraisal process described through the theory of persuasive hope. According to the theory, these messages acted as a stimulus to promote an opportunity to survive cancer, subsequently signalling an appraisal for a future outcome that was perceived as desirable, important, being possible, and consistent with their goals^31^. The appraisal would have triggered an optimistic sense of survival through screening, a hopeful aspiration largely expressed among our participants. This emotional response would have generated an approach tendency that stimulated intentions to initiate preparatory actions for achieving the desired outcome, likely expressed through empowering elements, such as being motivated and feeling confident to act^32^. However, it is important to be mindful of not overselling hope to the extent of compromising actual realities, which may be perceived as misleading as opined by a few participants. Messages that exaggerate the benefits or downplay the challenges of a recommended action can diminish a message’s overall effectiveness or deter individuals from taking action^33^.

Preference for fear appeal messages were mostly driven by a wide array of negative emotions that heightened awareness and perceived threats related to breast cancer. Although fear was the third most preferred appeal, the fear appeal messages may not have exerted their full potential when evaluated against established theoretical frameworks on effective fear communication. According to the Extended Parallel Process Model^34^, fear-inducing messages should elicit both threat and efficacy appraisals to influence behaviour. By standardising the length of experimented messages to provide minimal content aimed at highlighting each appeal in the present study, the fear messages presented facts about the dangers of breast cancer, but lacked information on the behavioural steps required to obtain screening, as well as an explanation of screening effectiveness in preventing late-stage breast cancer. Absence of such information limits adequate self-efficacy and response efficacy appraisals. Self-efficacy involves an individual’s assessment of how capable they can address a threat effectively^35^, whereas response efficacy concerns the perceived effectiveness of the recommended behaviour^34^. Without these efficacy components, the messages risk inducing defensive avoidance rather than danger control, which may have muted their perceived value and preference among participants. Hence, future fear appeal message development should combine high threat with strong efficacy components to maximise their persuasive impact.

Our social appeal messages leveraged on social elements that emphasised shared social responsibility, collective action, and community well-being. These values theoretically align with the preferences of a country with a collectivist culture such as Malaysia^36^, and thus should yield positive responses via these tested messages. Indeed, participants who preferred them expressed reasons that echo these promoted values. However, in totality, we observed a lukewarm reception to these messages.

Although we did not explore further, a possible reason for this observation could be due to the manner in which the experimental messages leveraged the influence of generic peers over a broad societal or nationalistic perspective to promote the advocated behaviour. Such framings may feel perceptually abstract and insufficiently relatable, thereby limiting persuasive impact. While these appeals highlight communal duty, they may be less effective if individuals are more influenced by specific important referents rather than an undifferentiated “society.” A potential direction for future work is to examine social appeals using a norms approach by applying descriptive norms that focus on relatable persona, or injunctive norms communicated by respected in-group authorities such as family elders or community leaders. These approaches may carry greater influence than generic peer references and have been found to be positively influential within collectivist cultural contexts^37,38^.

A second possible contributing factor relates to the study’s urban sample. According to the theory of social change and human development, urbanisation induces socio-cultural environmental pressures that potentially shift cultural values through gradual adaptation, making urban populations lean slightly toward individualism, even in collectivist societies^39^. Individualistic tendencies may have rendered the social appeal messages, which focus on communal benefit and collective responsibility, less appealing. Together, these factors may explain the tepid preference observed for the social appeal messages tested in this study.

Generally, responses from the shame-guilt and sadness appeal messages reflected a sense of moral obligation centred on preventing negative consequences from befalling loved ones. This insight supports conceptualising these two appeals under a broader moral appeal^40^. Individuals who likely exhibited collectivist cultural values appeared to appreciate the negative emotions that evoked a sense of responsibility to safeguard their loved ones^41^. However, participants who disliked these messages primarily criticised their negative framing and the involvement of loved ones. Interestingly, this reaction contrasts with responses to the fear appeal messages which, although also negatively framed, did not attract similar criticisms. A key distinction between these groups of messages is that the fear appeals directed negative implications towards the self, whereas the shame–guilt and sadness appeals highlighted the consequences for loved ones. The negatively framed emphasis on personal failure causing close others to suffer may have inadvertently induced discomfort and psychological reactance, rather than motivating action. This suggests that moral-based appeals need to be framed carefully. While concern for family is culturally salient in Malaysia, overly coercive or blame-oriented messaging risks backfiring and reducing message acceptance.

Due to its entertaining nature, incorporating humour into health communication has been suggested to be advantageous at drawing and holding attention, while helping to attenuate uncomfortable responses associated with certain health topics that may provoke anxiety, fear, or embarrassment^42^. While a few participants concurred with such advantages, our findings largely unveiled multiple negative externalities that occurred with the use of a humour appeal to promote breast cancer screening behaviour. These backfiring effects were reflective of factors cited from previous studies. Suka et al. explained that lower comprehensibility was a factor that caused a significant decline to the persuasiveness and acceptability of humour appeal messages that promoted cancer screening among Japanese participants^24^. A humour appeal, being unserious in nature, could also be perceived as a mismatch when used to convey a serious medical procedure, such as breast cancer screening^25^. McGraw et al. found that humorous public service announcements provided lesser motivation to search for health information because they downplayed the importance of the promoted issue^43^. The influence of culture is also likely possible. Eastern culture tends to view humour negatively compared to Western culture^44^, which may explain the heavy criticisms received for portraying a serious health issue in a humoristic manner. Our findings add on to the variable outcomes observed with humour based public health communication that promoted various health related topics such as organ donation and vaccination^45,46^. Such variabilities underscore the importance of exercising caution when integrating humour into public health messages.

### Limitations

Our study presents several limitations. Firstly, participants were asked to read through and carefully select their preferred and disliked messages, which likely promoted central or analytical route processing, as described in the Elaboration Likelihood Model^47^. Consequently, the findings primarily reflect participants’ cognitive preferences rather than the peripheral or heuristic responses that often guide behaviour through real-world health message exposure, where attention may be limited. This trade-off was necessary to collect rich qualitative insights, but it may limit the generalisability of our findings to everyday communication contexts.

Secondly, only reactions to exposed messages were studied in depth. Objective evaluations to measure the actual impact of messages on influencing intention or behaviour were outside the study’s scope. Thirdly, the tested messages were solely textual and consistent in length. This could have reduced the overall propensity to arouse emotions and the effectiveness of certain appeals which may require longer textual lengths, or have visual or auditory stimulations to exert their full potential. However, we used this approach to avoid confounding elements which may influence the choice of appeals made by participants under the supervision of individuals with health communication expertise.

Lastly, our sample consisted solely of urban Klang Valley residents. Their responses may not represent those of rural or lower-income groups, who differ in health literacy and message interpretation.

### Future directions

The acknowledged limitations suggest several future opportunities. First, messages delivered in brief, low-attention exposure formats, such as posters at bus stops or short social media clips, could be used to investigate the peripheral effects of the studied appeals. This would enable objective assessments on how public health messages that are incorporated with different appeals influence attitudes, intentions, and behaviours in a more naturalistic and generalisable setting.

Second, experimented messages could also be explored using multiple media formats, such as visuals, stories, and videos, and structured to reflect real-life campaigns, which would enhance ecological validity. Third, the effects of combining different appeals should be investigated to explore potential synergies that may amplify persuasive effects. Fourth, future work should include more diverse populations that extend beyond urbanised localities to improve generalisability of findings.

Fifth, Malaysia’s multi-ethnic society composition possesses a rich cultural diversity that may expose nuances towards how each major ethnic group responds to authoritative and emotional appeals. Thus, there are opportunities to explore from a quantitative design perspective, cross-cultural patterns that may exist when individuals respond to messages with authoritative and various emotional appeals.

Finally, future research could employ experimental or longitudinal designs to examine how the investigated appeals actually influence real-world screening uptake.

## Conclusion

Hope and fear appeals hold promising potential towards motivating breast cancer screening among Malaysian women. Authoritative messages with a tailoring component are also equally promising, despite the lack of an emotional element. Hence, these appeals should be further explored, both independently and in combination, to investigate their potential in influencing actual breast cancer screening behaviour among Malaysian women through appropriate public health communication strategies. Given that messages employing certain appeals significantly influences the motivation of women bidirectionally, pre-testing public health messages is strongly recommended to avoid unwanted resistance or reactance from the public during mass implementation.

## Supplementary Information

Below is the link to the electronic supplementary material.

Supplementary Material 1

## Data Availability

Transcripts used for this study belongs to the Malaysian Ministry of Health. These transcripts may be available from the corresponding author via a formal request through relevant authorities at the Malaysian Ministry of Health.
