# Supplementary material for: A qualitative study exploring Malaysian women’s preferences for authority or emotional appeals in public health messages promoting breast cancer screening
Source: Sci Rep. 2026 Jan 12;16:1312. doi: 10.1038/s41598-025-31687-7 (PMC12796188; doi:10.1038/s41598-025-31687-7)
Supplement: Supplementary file 1 — Supplementary Material 1 [file 41598_2025_31687_MOESM1_ESM.docx]

**Supplementary material**

**A Qualitative Study Exploring Malaysian Women’s Preferences for Authority or Emotional Appeals in Public Health Messages Promoting Breast Cancer Screening**

**METHODS**

**Study Procedure**

NYLH, PXK, CEL, KP, ANMN, and WYH conducted in-depth interviews on identified participants. The interviewers had backgrounds in healthcare or health education; four are qualified and have experience to conduct interviews while two are new to qualitative research. However, they were trained by one of the experienced interviewers. All interviewers attended a formal workshop to standardise the understanding and method of interviewing with the instruments. Reflexivity was ensured through regular team discussions during data collection, which allowed for critical examination of responses and consistency in the interview approach.

Participants were briefed on the study procedure and written informed consent was obtained before the interview commenced. Participants were then given the 14 investigated messages in a random order and provided sufficient time to read them through. After that, participants were prompted to select messages that they liked or found motivating for attending breast cancer screening. A message that was selected by a participant counted as one vote of preference for that message. This selection exercise was integrated into the interview process as a structured elicitation technique to support participants’ reflection and to spark deeper discussion only towards messages which stood out.

For each selected message, participants were asked if it evoked any form of emotions before asking them to freely express reasons that led them to shortlist the message. This entire process was repeated for messages that participants disliked or felt demotivating.

All interviews were recorded using an audio recorder. Periodic data consolidation was conducted, and participant recruitment was halted upon reaching saturation. Data collection period lasted for three months, i.e. July to October 2023.

**RESULTS**

**Table S1:** Tabulation of preferred and disliked appeals among interviewed participants.

a. Combined number of votes for both messages in each appeal.

b. Number of votes for an appeal after disregarding combined votes that are doubled for each appeal.

**Section 1: Themes and subthemes emerging from preferred message appeals**

**1.0 Theme: Effective Message Construct**

This theme highlights the structural and linguistic elements of a message that enhance its effectiveness in clearly conveying the intended meaning to the target audience.

- 1. **Easy to comprehend**

This subtheme captures expressions regarding the message construction characteristics being clear, simple and direct to the point. It highlights how the use of simple language, clear sentence structure, easily interpretable content, and numeric or simple statistics, ensures effective communication with the target audience.

**2.0 Theme: Relatable Communication**

This theme highlights the importance of messages which are aligned with specific characteristics, experiences, and contexts that are relevant to the audience to elicit favourable responses, subsequently enhancing audience engagement and resonance.

**2.1 Age-relevant communication**

This subtheme illustrates the effect of tailoring messages to align with the target age group of the intended audience. By using age-specific language or highlighting a particular age group, constructed messages will feel personally meaningful to the intended audience, which helps to encourage the desired response.

**2.2 Relatable experience**

This subtheme categorises responses associated with messages communicating content that are relatable to an audience’s life experience. Messages that reflect real-life situations triggers a recall of familiar experiences that were personal or within one's social circle, which enhances resonance, emotional engagement, and motivation to act.

**3.0 Theme: Empowerment**

This theme captures responses from individuals who expressed that the messages inspired positive reflections, triggering thought processes that encouraged them to take control of their health. These reflections fostered a sense of empowerment, motivating them to develop intentions or initiate proactive decisions to screen for breast cancer.

**3.1 Awareness raising**

The role of information to raise awareness among individuals to make informed decisions was captured through this subtheme. Awareness can be raised through providing clear and relevant knowledge regarding the subject of interest. This includes information about the health risks, health consequences, and available preventive measures which individuals can adopt.

**3.2 Sense of responsibility**

This subtheme highlights how individuals feel responsible to safeguard their health, either for their own well-being or to care for their loved ones. This sense of duty encourages proactive health behaviours to take preventive actions.

**3.3 Sense of urgency**

This subtheme describes the immediate need to adopt a health behaviour in response to triggers within exposed messages. These triggers activate cognitive processes which compel individuals to take prompt preventive actions based on certain informational cues.

**3.4 Motivational**

This subtheme highlights how messages can inspire and encourage individuals to take proactive health actions.

**3.5 Invoking confidence**

This subtheme describes how messages can instil a sense of capability in individuals to take proactive health actions. By using reassuring language or positively framed messaging, individuals feel confidently empowered to make informed health decisions.

**3.6 Self-Preservation**

This subtheme focuses on the instinctual drive by individuals to protect their life and well-being by taking proactive measures related to health-related decisions. Such expressions arise from how messages depict how one’s life is associated with the act of adopting a health behaviour.

1. **Theme: Obligation to Loved Ones**

This theme reflects on the idea that participants are motivated to take preventive actions by adopting a health behaviour, not just for personal benefit, but to also protect their loved ones.

**4.1 Preventing emotional burden**

This subtheme highlights how the desire to avoid their loved ones from experiencing emotional distress serves as a strong motivator for adopting preventive health behaviours.

**4.2 Prioritising family well-being**

This subtheme highlights the motivation by individuals to adopt preventive health behaviours for the sake of their family's well-being. It reflects the belief that staying healthy is not just a personal responsibility but also a way to support and protect their loved ones.

**4.3 Avoiding shame through early action**

This subtheme reflects the perception that individuals are motivated to take proactive health measures early as a demonstration of responsibility, aiming to avoid potential judgment from family members or loved ones.

**5.0 Theme: Fear Arousal**

This theme examines how messages invoked fear to promote an emotional response that motivates preventive actions. By highlighting potential risks or negative consequences of a disease, the message triggers a fear response to encourage the promoted health behaviour.

**5.1 Avoidance of negative outcomes**

This subtheme captures responses on how messages which arouse fear by highlighting potential negative outcomes motivate individuals to take preventive health actions to avoid those undesirable consequences.

**5.2 Heightened perceived susceptibility**

Fear can be aroused via a heightened awareness of an individual’s personal risk of facing a particular health issue. Emphasising the likelihood of being affected by a disease increases concern and drives individuals to adopt preventive health behaviours to protect themselves.

**6.0 Theme: Authoritative Voice from a Reputable Source**

This theme explores how a credible and authoritative source can significantly impact the decision or perception of individuals towards a prescribed health-related behaviour.

**6.1 Amplified trust and credibility**

This subtheme emphasises the use of a credible source in a message to strengthen its impact and enhances trustworthiness of the shared information.

**6.2 Perceived government care**

This subtheme describes how messages that feature government agencies providing authoritative health advice reinforces the perception that the government is genuinely committed to the well-being of the population.

**7.0 Theme: Survival Optimism**

This theme highlights responses regarding the positive consequences of increasing the chances of survival through adopting a preventive health behaviour. This sense of optimism motivates individuals to engage in the recommended health behaviour.

**8.0 Theme: Social Cohesiveness**

This theme focuses on the sense of unity and shared responsibility among members of a community. It captures the importance of collective action where individuals feel connected with each other while working towards a common societal goal.

**8.1 Advocacy as a social responsibility**

This subtheme emphasises the role of individuals to advocate for a health behaviour within their communities by conferring a sense of collective responsibility to educate and encourage community members.

**8.2 Role modelling**

This subtheme highlights the idea of individuals being role models to inspire women around them to follow their lead in adopting a specific health behaviour.

**8.3 Solidarity**

This subtheme describes the sense of unity and mutual support within a community to achieve a common goal of encouraging a health behaviour for the greater good.

**8.4 Desire for community wellbeing**

This subtheme reflects on aspirations to foster a healthy community in the entire country through encouraging a health behaviour.

**9.0 Theme: Engagement through Humour**

This theme highlights how humour messages create engagement and receptiveness. Humour helps to capture the audience’s attention while conveying content in a light-hearted manner to encourage a positive response.

**9.1 Approachable**

This subtheme focuses on how messages with a light-hearted tone makes them more likable and easier to engage with. Humour makes a depressing health topic feel less intimidating, which helps to reduce negative emotions and renders the content of the health message more accessible.

**9.2 Unique presentation**

This subtheme captures the unique method of presenting information in an unconventional or creative manner. By deviating from traditional formats, the message becomes more engaging and memorable, leading to greater receptiveness from the audience.

**Section 2: Themes and subthemes emerging from disliked message appeals**

1. **Theme: Ineffective Communication**

This theme encompasses insights highlighting messages that failed to capture attention, resonate, persuade, or effectively convey its intended meaning. These failures are based on various limitations inherent in a message’s construct or characteristic, such as its tone, relatability, clarity, comprehensibility, or presentation manner. These factors lead to disinterest, confusion, or rejection by the audience.

**1.1 Unappealing message construct**

This subtheme captures feedback related to messages being structured or phrased in a manner that was either perceived as boring, too formal, or too commonly used. These responses infer how information becomes less persuasive, memorable or impactful if it is conveyed in an unappealing structure.

**1.2 Unemotional**

This subtheme highlights how messages are perceived as less engaging and persuasive because they fail to evoke an emotional response.

**1.3 Misleading**

This subtheme describes messages being misleading by providing information which are deemed inaccurate, or do not reflect reality as experienced in the real-world.

**1.4 Indirect messaging**

This subtheme captures how messages fail to convey their intended meaning due to implicitly communicating information. Presenting content that requires interpretation to grasp an intended meaning can reduce clarity or risk misinterpretation.

**1.5 Illogical messaging**

This subtheme emphasises the perception of messages being illogical for not following a rational flow or sensible reasoning.

**1.6 Message misinterpretation**

This subtheme captures instances where messages were misinterpreted, diverting the audience from their intended meaning. This results in confusion or unintended reactions.

**1.7 Redundant messaging**

This subtheme captures sentiments about messages that fail to offer new insights or merely emphasise obvious points, making them sound unpersuasive.

**1.8 Negativity**

This subtheme captures perceptions of messages that are being overly negative. Framing messages with negative elements such as sadness, death, fear, regret, guilt, or shame, causes messages to be unattractive and lead to resistance or disengagement from the intended health behaviour.

**1.9 Not relatable**

This subtheme captures the expressions of how messages were perceived as being unrelatable due to the lack of personal relevance or connection to the audience’s own experiences.

**1.10 Inability to decipher numbers**

This subtheme conveys the expression of being unattracted to messages which use numbers due to difficulties relating or appreciating the quantity that the message was attempting to picture.

- 1. **Challenges in comprehension**

This subtheme captures expressions of messages being difficult to understand or interpret due to the way that the message is worded or structured, leading to a failure in conveying the intended meaning.

**2.0 Theme: Inappropriate Communication**

This theme focuses on messages being inappropriate for the target audience, as it may draw negative connotations or feel insensitive. Inappropriate messages may offend people and cause them to resent or disregard the information conveyed.

**2.1 Trivialising serious issues**

This subtheme captures comments about messages that are perceived to downplay the seriousness of a health issue, thus making them feel insignificant or less urgent, which may subsequently reduce motivation to take action.

**2.2 Provocative**

This subtheme is based on criticisms of messages that are perceived as insensitive or confrontational, potentially provoking negative emotional reactions such as discomfort, resistance, irritation, or anger.

**2.3 Age-Inappropriate messaging**

This subtheme captures concerns about messages being perceived as unsuitable for certain age groups due to factors such as relevance, tone, or approach, which may affect how the content is received and interpreted.

**3.0 Theme: Perceived Coercion**

This theme captures the perception that messages evoking a sense of pressure or obligation may lead to resistance or negative emotional reactions, which reduces their effectiveness in encouraging a health behaviour.
